# Nutraceutical Improvement Increases the Protective Activity of Broccoli Sprout Juice in a Human Intestinal Cell Model of Gut Inflammation

**DOI:** 10.3390/ph9030048

**Published:** 2016-08-12

**Authors:** Simonetta Ferruzza, Fausta Natella, Giulia Ranaldi, Chiara Murgia, Carlotta Rossi, Kajetan Trošt, Fulvio Mattivi, Mirella Nardini, Mariateresa Maldini, Anna Maria Giusti, Elisabetta Moneta, Cristina Scaccini, Yula Sambuy, Giorgio Morelli, Simona Baima

**Affiliations:** 1 Food and Nutrition Research Centre, Consiglio per la ricerca in agricoltura e l’analisi dell’economia agraria, Via Ardeatina 546, 00178 Rome, Italy; simonetta.ferruzza@crea.gov.it (S.F.); fausta.natella@crea.gov.it (F.N.); giulia.ranaldi@crea.gov.it (G.R.); chiara.murgia@monash.edu (C.M.); carlottarossi22@hotmail.com (C.R.); mirella.nardini@crea.gov.it (M.N.); mtmaldini@uniss.it (M.M.); elisabetta.moneta@crea.gov.it (E.M.); cristina.scaccini@crea.gov.it (C.S.); yula.sambuy@crea.gov.it (Y.S.); giorgio.morelli@crea.gov.it (G.M.); 2 Food Quality and Nutrition Department, Research and Innovation Centre, Fondazione Edmund Mach, Via Edmund Mach 1, 38010 San Michele all’Adige (TN), Italy; kajo@wajdusna.net (K.T.); fulvio.mattivi@fmach.it (F.M.); 3 Department of Experimental Medicine, Section of Medical Physiopathology, Food Science and Endocrinology, Sapienza University, P.le Aldo Moro 5, 00185 Rome, Italy; annamaria.giusti@uniroma1.it

**Keywords:** functional food, broccoli sprouts, Caco-2, intestinal permeability, inflammatory response, bioactive molecules, phenolic compounds, anthocyanins, sulforafane

## Abstract

Benefits to health from a high consumption of fruits and vegetables are well established and have been attributed to bioactive secondary metabolites present in edible plants. However, the effects of specific health-related phytochemicals within a complex food matrix are difficult to assess. In an attempt to address this problem, we have used elicitation to improve the nutraceutical content of seedlings of *Brassica oleracea* grown under controlled conditions. Analysis, by LC-MS, of the glucosinolate, isothiocyanate and phenolic compound content of juices obtained from sprouts indicated that elicitation induces an enrichment of several phenolics, particularly of the anthocyanin fraction. To test the biological activity of basal and enriched juices we took advantage of a recently developed in vitro model of inflamed human intestinal epithelium. Both sprouts’ juices protected intestinal barrier integrity in Caco-2 cells exposed to tumor necrosis factor α under marginal zinc deprivation, with the enriched juice showing higher protection. Multivariate regression analysis indicated that the extent of rescue from stress-induced epithelial dysfunction correlated with the composition in bioactive molecules of the juices and, in particular, with a group of phenolic compounds, including several anthocyanins, quercetin-3-Glc, cryptochlorogenic, neochlorogenic and cinnamic acids.

## 1. Introduction

Epidemiological data indicate that frequent fruit and vegetable consumption is associated with a reduction in morbidity and mortality from cardiovascular diseases, type 2 diabetes mellitus and several cancers [1,2,3,4], and this effect has been attributed to their high content in vitamins, minerals, polyphenols and other bioactive molecules. Indeed, several isolated phytochemicals have been shown to exert a potential health-promoting effect in different experimental systems [5,6,7]. However, studies with whole foods, rather than with isolated bioactive chemicals, are strongly recommended to account for the effects of possible interactions of different molecules within the context of the food matrix [1,8]. A common drawback of this type of study is the poor chemical characterization and the great qualitative and quantitative compositional variability of vegetable foods [8,9]. In fact, the metabolite content of a given fruit or vegetable depends on many different factors such as cultivar, geographical origin, agronomical practice, storage, processing and preparation. As sessile organisms, plants have evolved a high metabolic plasticity as a sophisticated adaptive defensive response mechanism to grow and survive under biotic and abiotic stresses [10,11]. While this flexibility contributes to the compositional variability that challenges the scientific assessment of the benefits of plant foods, the environmental response of plants can be exploited to enhance the content of specific health-related molecules, or classes of molecules, within the food matrix. In fact, most of the bioactives that have been shown to exert a protective role in animals are secondary metabolites, mainly phenolics, that plants synthetize to protect themselves from adverse environmental conditions. Therefore, elicitation, i.e., manipulation of growth conditions to trigger the synthesis and accumulation of bioactive secondary metabolites, can be considered a good strategy for functional enrichment of plant-derived foods [12]. Recently, by systematic evaluation of many different elicitors, we have identified sucrose as the elicitor that provides the most significant overall effect on phytochemical composition of young broccoli seedlings [13].

Broccoli, along with many other common vegetables such as cauliflower, kale, brussels sprouts etc. belonging to the *Brassicaceae* family, are considered to have a high health-promoting potential for their richness in vitamins, minerals, fibers, and bioactive secondary metabolites [14,15]. Among these, considerable attention has been given to glucosinolates (GLSs), a class of secondary metabolites synthetized almost exclusively by this plant family, and, in particular, to their hydrolysis products’ isothiocyanates (ITCs) for their ability to induce phase II detoxification enzyme activities and anticancer potential [16,17]. Sprouts of *Brassicaceae* contain higher levels of phenolic compounds and GLSs than adult plants [18,19,20], and are increasingly becoming popular as natural functional foods.

Critical for the assessment of potentially health-related bioactivity of conventional or functionally enriched food extracts is the choice of a physiologically relevant experimental system and of appropriate endpoint(s) [8,21]. Although epidemiologic and intervention studies are absolutely required for evaluation of functional foods, it is generally recognized that properly designed animal and cell culture studies can help to elucidate mechanistic aspects of food/food component effects [8,21].

The aim of this study was to functionally test the effects of phytochemical enrichment of broccoli sprouts elicited with sucrose on an intestinal cell model of gut inflammation. Since several bioactive secondary metabolites from *Brassicaceae* are reported to possess anti-inflammatory properties [22,23,24], we took advantage of a recently developed model of inflammatory stress in the human intestinal Caco-2 cell line [25] to compare the biological activity of juices, from both basal and elicited broccoli sprouts, characterized by different polyphenol, flavonoid and anthocyanin profiles. Pre-incubation of differentiated intestinal Caco-2 cells with these juices conferred protection from the inflammatory stress induced by tumor necrosis factor α (TNFα) under marginal zinc deficiency. A chemometric approach was used to correlate the protective effect of broccoli juices with their content in bioactives, detecting a significant association with the composition in anthocyanins and other polyphenolic compounds.

## 2. Results

### 2.1. Preparation of Basal and Enriched Broccoli Sprout Juices

**Figure 1:**
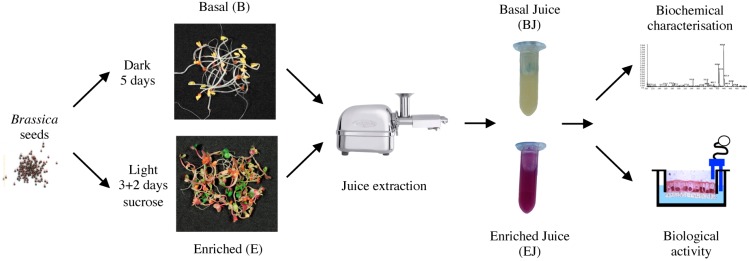
Assessment of growth conditions on composition and biological activity of broccoli sprouts. Schematic representation of the experimental workflow used to evaluate and compare the composition and the biological activity of juices prepared from broccoli sprouts grown under two different conditions.

In this study, young broccoli seedlings (sprouts) were grown in a climatic chamber in which growth conditions can be easily controlled and modified. Biomass yield was higher for sprouts grown in basal condition (B) than for those in enriching condition (E) (12 g of seeds produced 116.26 ± 8.58 g and 86.69 ± 11.51 g of sprouts, respectively; mean ± SD). The sprouts grown in the two conditions were morphologically different: elongated with yellowish, unexpanded cotyledons in the case of B and short with expanded green and reddish cotyledon in the case of E (Figure 1). Immediately after harvesting, aqueous juices were prepared from sprouts with a slow rotating screw (cold press) as it had previously been shown that, when used to prepare juices from adult broccoli, it resulted in maximal recovery of polyphenols and in highest growth inhibitory effects on cancer cells, compared to centrifugal juicer or blender [26]. Aliquots of the juices were used for compositional analysis and functional testing on cell cultures (Figure 1).

### 2.2. Compositional Analysis

**Figure 2:**
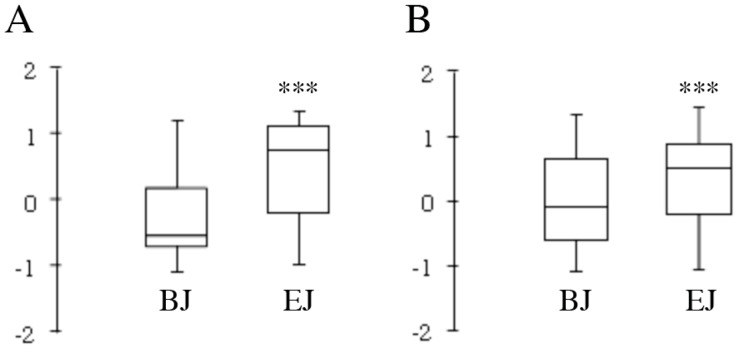
Box plot of LC-MS untargeted metabolomic fingerprinting. Distribution of LC-MS normalized (Z-scores) data from three biological replicates of juice from sprouts grown under two experimental conditions (basal, BJ; enriching, EJ). (**A**) Negative ion mode (mean values of *n* = 598 peaks); (**B**) Positive ion mode (mean values of *n* = 855 peaks). Center line shows the median; box limits indicate the 25th and 75th percentiles; whiskers extend to the most extreme data point within 1.5 times the interquartile range. *** *p* < 0.0001 EJ vs. BJ.

To characterize the phytochemical diversity induced by different growth conditions, LC-MS metabolomic fingerprinting data were used as descriptors of the composition of the broccoli sprout juices. Untargeted LC-MS metabolomic profiling analysis was carried out on three independent biological replicates of each type of juice in both negative and positive ion modes, yielding 598 and 855 features, respectively. Following the automated data mining and pre-treatment step of LC-MS metabolomic data, the intensity value of each anonymous peak was normalized by Z-score transformation. The datasets of the normalized variables were graphically visualized in Figure 2. Statistical analysis of data distribution confirmed that the composition of the juices obtained from sprouts grown under basal (BJ) and enriching conditions (EJ) was significantly different (*p* < 0.0001). Targeted LC-MS analysis was then applied to compare the composition of BJ and EJ with respect to phytochemicals that can potentially affect the biological activity of sprouts (Table 1).

Sulforaphane (SFN), the major inducer of phase II enzymes, and SFN-nitrile, both derived from enzymatic conversion of glucoraphanin (the most abundant GLS present in *B. oleracea* sprouts [27]), were detected at similar levels in both BJ and EJ (Table 1). Glucoraphanin and other GLSs were not present in both BJ and EJ, as expected from myrosinase activation during juice preparation. The total content of different classes of phenolic compounds was found to be higher in EJ than in BJ, with a dramatic increase of the anthocyanin fraction (total polyphenols (TP) 1.6-fold, total flavonoids (TF) 2.6-fold, and total anthocyanins (TA) about 10-fold enrichment, respectively). Targeted LC-MS analysis allowed the detection and identification of 18 phenolic compounds and 14 anthocyanins (Table 1). The vast majority of the 18 polyphenols detected were found significantly increased in EJ. In particular, a dramatic difference was observed for neochlorogenic acid, with a concentration about 50 times higher, and for chlorogenic and caffeic acid, that both showed a 13-fold enrichment compared to BJ. Only four phenolic compounds, namely sinapic acid, syringic acid, 4-aminobenzoic acid, and syringaldehyde, showed an inverse trend, being significantly more abundant in BJ than in EJ. Among the 14 anthocyanins detected in EJ, only cyanidine-3-diglucoside-5-glucoside (Cy-3-dGlc-5-Glc) was found also in BJ, while the others were below detection level (Table 1).

### 2.3. In Vitro Testing of Biological Effects

The gastro-intestinal mucosa is a selective barrier that regulates compounds’ bioavailability and has a pivotal role in the interaction between food components and the organism. Thus, an intestinal cell model seems an appropriate choice to investigate in vitro the potentially beneficial effects of plant food preparations and the underlying molecular mechanisms. Upon differentiation on filter inserts, human intestinal Caco-2 cells form a polarized monolayer that separates two distinct compartments, corresponding in vivo to the intestinal lumen and the blood circulation, and display many of the physiological and morphological characteristics of intestinal absorptive enterocytes [28,29,30,31]. Addition of the food extract of interest to the apical (AP) compartment of this cell model challenged with pathological or toxic stimuli allows the study of the protective capacity of the food/food components under investigation [32,33].

**Figure 3:**
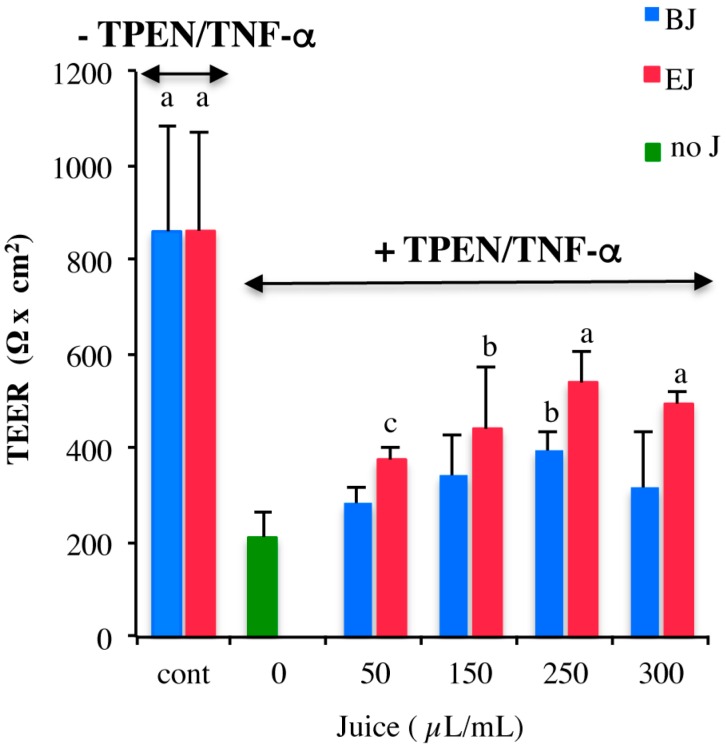
Broccoli sprout juices protect epithelial barrier integrity of zinc-depleted Caco-2 cells exposed to TNFα. Caco-2 cells differentiated on filters for 21 days were pre-incubated for 14 h with experimental medium containing the indicated amount of broccoli juice in the AP compartment (red bars: EJ; blue bars: BJ; green bar: experimental medium without juice addition, noJ). Cells were then zinc deprived by TPEN incubation and exposed to TNFα for 5 h. As control, a set of filters was pre-incubated with the addition of 250 μL/mL of BJ or EJ for 14 h and then maintained in experimental medium for the whole experiment. TEER values, measured at the end of the experiment, are expressed as means ± SD from three experiments performed in triplicate. Statistical analysis was performed by one-way ANOVA followed by Fischer post hoc test. Different letters above bars indicate significant differences vs. TPEN/TNFα-treated cells without juice pre-treatement (green bar). a: *p* < 0.0001; b: *p* < 0.01; c: *p* < 0.05.

We have recently demonstrated that Caco-2 cells with marginal zinc deficiency respond to the inflammatory cytokine TNFα undergoing a gradual increase in cell monolayer permeability that can easily be monitored by measuring the decrease in trans-epithelial electrical resistance (TEER) [25], a characteristic parameter of epithelial cell layers, reflecting the functionality of the tight junctions and the integrity of the cell monolayer. A decrease in TEER values is indicative of early sub-lethal cell toxicity [34,35]. This experimental model was exploited to assay the biological effects of broccoli sprouts’ juices. Filter grown, differentiated Caco-2 cells were pre-incubated from the AP side for 14 h in medium containing different dilutions of BJ or EJ. After removal of juice-containing medium, cells were marginally zinc-depleted by incubation for 2 h with *N*,*N*,*N*′,*N*′-tetrakis(2-pyridylmethyl)ethane-1,2-diamine (TPEN) and subsequently challenged with TNFα for 5 h (TPEN/TNFα treatment). As indicated in Figure 3, pretreatment with EJ, at all dilutions tested, determined a significant protection from the effects of TPEN/TNFα on TEER with respect to cells not exposed to the juice. The extent of protection was correlated to the amount of juice added and reached its maximum at a dilution of 250 μL/mL. Pretreatment with BJ always resulted in lower effect compared to EJ and a significant protection was only observed at 250 μL/mL. Thus, further experiments were conducted using juice pre-incubation at 250 μL/mL. It is noteworthy that pre-treatment of cells with medium containing the same dilution of juices had no effect on TEER, indicating that both BJ and EJ alone did not affect epithelial integrity (Figure 3).

**Figure 4:**
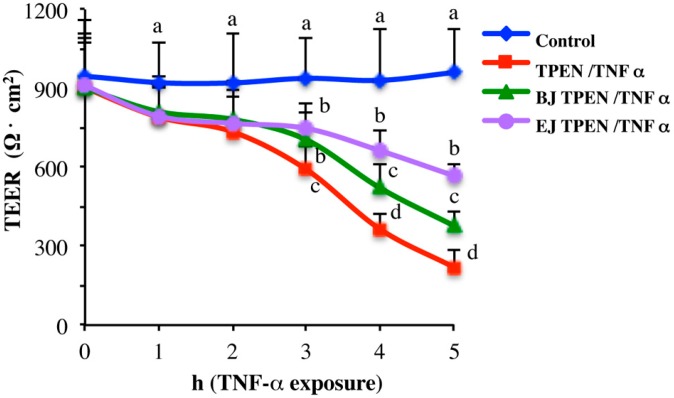
Time course of the protective effects of broccoli sprout juices in TPEN/TNFα treated Caco-2 cells. Caco-2 cells differentiated on filters for 21 days were pre-incubated for 14 h in experimental medium with BJ (green triangles), EJ (purple circles) or without juice (red squares) addition in the AP compartment. Cells were zinc deprived by TPEN incubation and exposed to TNFα except untreated control cells (blue diamonds) that were maintained in experimental medium throughout the experiment. TEER was monitored in the last 5 h of the experiment during TNFα treatment. Data are the mean ± SD from three experiments performed in triplicate. One-way ANOVA was performed on mean-centered data followed by Fischer post hoc test. Different letters above bars indicate significant differences (*p* < 0.05) among treatments.

To compare the protective effects of BJ and EJ, time-course experiments were carried out, monitoring TEER values over 5 h of TNFα incubation. As shown in Figure 4, the protective ability of sprout juices was already significant after 3 h of TNFα exposure. However, at this time point, BJ and EJ displayed comparable protection, while at later times, EJ protection was significantly higher compared to that of BJ.

**Figure 5:**
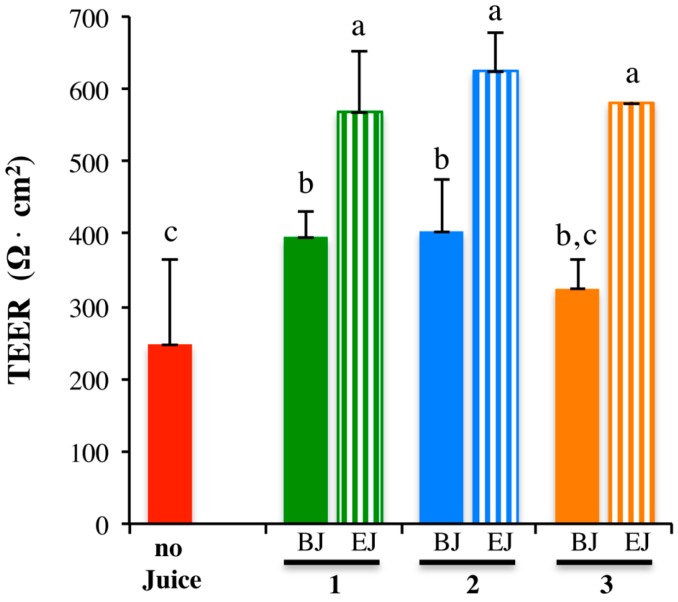
Juices from independendent sprouts growths show similar effects in TPEN/TNFα treated Caco-2 cells. Caco-2 cells differentiated on filters for 21 days were pre-incubated for 14 h in experimental medium with BJ (full bars) or EJ (striped bars) obtained from three indipendent sprouts growths (green, blue and orange bars) or without juice (red bar) addition in the AP compartment. Cells were then zinc deprived by TPEN incubation and exposed to TNFα. TEER values were measured after 5 h of TNFα treatment. Statistical analysis was performed by one-way ANOVA followed by Fischer post hoc test. Different letters above bars indicate significant differences (*p* < 0.01) among treatments.

To determine the reproducibility of the biological effects of the juices, three different batches of BJ and EJ (namely Juices 1, 2 and 3), prepared from independent sprout growths, were analyzed. As shown in Figure 5, all three batches of EJ, after 5 h of treatment, conferred a similar protection in preventing TEER reduction produced by TPEN/TNFα inflammatory stimulus. All three BJ protected significantly less than their corresponding EJ.

### 2.4. Multivariate Analysis of Composition and Correlation with the Biological Effect

**Figure 6:**
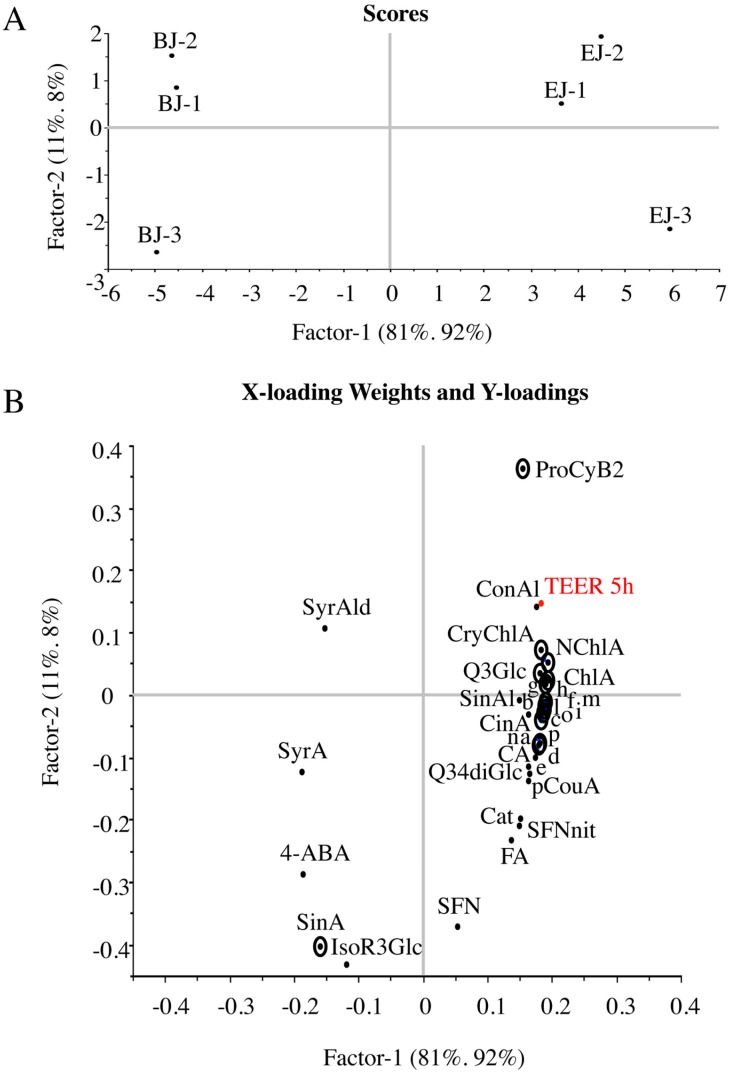
TPEN/TNFα-treated Caco-2 cell protection is correlated to phytochemical enrichment of broccoli sprout juice. Partial least square (PLS) regression analysis was used to predict the biological effect exerted on Caco-2 intestinal cells exposed to TPEN/TNFα from composition data (see Table 1) of six juice samples corresponding to three independent replicates of sprouts grown under two different conditions. (**A**) Scores plot showing the distribution of juice samples: BJ-1 to 3, juices from sprouts grown in basal conditions; EJ-1 to 3, juices from sprouts grown in enriching conditions; (**B**) Loadings plot showing the correlation between phytochemical descriptors (X-variables) and biological effect (Y-variable). The secondary metabolites that significantly affect response prediction are circled.

To explore the relationship between the protective effect on Caco-2 cells subjected to inflammatory stimulus and the enrichment in bioactive phytochemicals, multivariate data analysis was performed, aimed at identifying the key drivers of the observed differences between BJ and EJ. To achieve this, Partial Least Square Regression (PLSR) was applied to model the relationship between quantitative data for the single phytochemicals listed in Table 1, as independent predictive variables (X), and the protective effect obtained in our cellular model, expressed as TEER at 5 h, as dependent variables (Y). As reported in Table 2, the first two dimensions of the built PLSR model could explain 100% of the variance for the cell protection attribute, namely TEER, using 92% of phytochemical content information. The first two principal components are illustrated in Figure 6. Notably, the 1st component (Factor-1 = 81%) explained 92% of the biological effect of the samples, while the 2nd component (Factor-2 = 11%) explained only a residual 8% of the biological effect. Juice samples were grouped on the plane according to sprout growth conditions. A clear separation was obtained along the first dimension, with samples from dark grown sprouts (BJ) positioned in the left quadrants, and samples from elicited sprouts (EJ) located in the right quadrants, while distribution of the samples on the second dimension (accounting only for 11% of the variance), indicated a residual limited biological variance between juices obtained from independent growths (Figure 6A). Interestingly, most of the phytochemicals measured were positioned on the right side of the plot contributing to the EJ description, except for sinapic acid, syringic acid, 4-aminobenzoic acid, syringaldehyde and isorhamnetin-3-glucoside, which were associated with BJ. The high weighted regression coefficients (BW) obtained from the estimated PLSR model identified the phytochemicals that significantly contributed to the differences observed in cell-protection effects (Table 2, Figure 6B). In particular, intestinal cell protection was positively correlated with procyanidin B2, cryptochlorogenic acid, neochlorogenic acid, quercetin-3-glucoside, cinnamic acid and five different cyanidine-3-glucoside, which could explain the correspondingly higher protective capacity of EJ compared to BJ, while a negative correlation was observed only with sinapic acid. The model was validated by full cross-validation. Pearson correlation coefficient and root mean square error for both calibration and validation of the model are shown in Table 2.

## 3. Discussion

*Brassicaceae* are a good source of many phytochemicals with health-related activity, and dietary consumption of *Brassica* vegetables has been associated with a reduction in the incidence of several pathological conditions including cancers and several chronic inflammatory diseases [23,36,37,38]. Both the profile and the amount of these phytochemicals are strongly affected by the genotype (different species/varieties) but also by environmental conditions during plant growth. It has been shown in different *Brassicaeae* that several nutrients [39], bioactives [27], and antioxidant capacity [40] are higher in sprouts grown in the light compared to those grown in the dark. Furthermore, the application of biotic and abiotic stress factors during growth, such as extreme temperatures, saline or osmotic stress, elicitors or hormones involved in the plant defense response, further increases the content of bioactive molecules, including GLSs, vitamins and phenolics [41,42,43]. In this work, sprouts where chosen as they are naturally enriched in bioactive molecules compared to the corresponding mature vegetable [18], they can be grown under fully controlled environmental conditions, and can easily be treated with elicitors to deliberately and specifically modify their phytochemical content. We found that treatment with sucrose that had been previously reported to elicit the accumulation of glucosinolate and phenolic compounds and to trigger the synthesis of anthocyanins in broccoli sprouts [43,44,45] provides the most significant overall effect on phytochemical composition of broccoli sprouts [13].

In this study, we aimed to evaluate the potential improvement of health-related biological activities associated with the compositional changes induced in broccoli sprouts by elicitation with sucrose. The assessment of the health-related effects of conventional, organic or novel foods is a very difficult and controversial task. Most studies suggesting beneficial effects of vegetable consumption have been performed with isolated phytochemicals, while it is increasingly accepted that it is the complex mixture of different nutrients and bioactive components acting on many different targets that make food, rather than single nutrients/bioactives, effective in reducing the risk of developing chronic and degenerative diseases and cancer [46,47]. In addition, cumulative and synergic effects of nutrient and bioactive secondary metabolites have frequently been reported and need to be taken into account [48]. Whether using in vitro, animal or human intervention studies [9,21], it is generally recognized that analytical description of the administered food is a prerequisite for any attempt at evaluating the health-related potential of a given vegetable or fruit. In fact, as plant food composition is strongly affected by many different factors, poor characterization of the food source and composition often weakens the conclusions drawn from published studies [9,21].

We have used an aqueous juice obtained from sprouts grown from the same seeds under highly controlled conditions, in order to obtain two different but well-defined and reproducible profiles in certain classes of phytochemicals. Metabolic fingerprinting of juices by targeted and untargeted metabolomics confirmed that the growth conditions applied determined both qualitative and quantitative changes in the molecular phenotype of basal and enriched juices. In particular, we observed that EJ was highly enriched in anthocyanins (as also shown by the color of the juice) and contained significantly higher levels of 14 phenolic acids and flavonoids with respect to BJ. This observation is in agreement with the previously reported induction of total phenolic compounds and anthocyanins in sprouts treated with sucrose [13,44,45]. An inverse trend was observed only for four phenolics (sinapic acid, syringic acid, 4-aminobenzoic acid, and syringaldehyde). Despite the reduction in EJ, sinapic acid represented the major phenolic compound in both BJ and EJ, confirming its predominance in *Brassica* [49,50]. All the anthocyanins found belonged to the cyanidin group and, except for the non-acylated Cy-3-dGlc-5-Glc, presented one or two aromatic groups (sinapic, p-coumaric, ferulic or caffeic acid) conjugated to the C3 sugar. In addition, half of them presented an aliphatic malonyl group attached to the C5 sugar. Remarkably, a conspicuous fraction of the anthocyanins in EJ were acylated with a synapoyl group, suggesting that free sinapic acid decrease in EJ might have been due to its conjugation to newly synthetized anthocyanins upon sucrose-induction. Although a similar anthocyanin pattern has previously been reported for *Brassicaceae*, some compounds were, to the best of our knowledge, identified for the first time in broccoli sprouts [51,52]. The increased stability of acylated anthocyanins [53,54] and their potential anti-hyperglycemic effect due to inhibition of α-amylase activity [55] make these compounds very attractive for food quality-improvement applications.

Health-promoting foods should enhance the ability of the organism to preserve homeostasis in order to cope with environmental changes and stresses that continuously challenge its functional equilibrium. Thus experimental models aimed at the assessment of the biological activity and the potential protective effects of food or food extracts should reproduce an imbalanced physiological state that can be modulated by the addition of the food or food extract under investigation. A protective role of food bioactives has been reported in the physiological regulation of the inflammatory processes that are involved in the onset of several chronic pathologies [1,16,56]. Cultured cell models (monocytes, macrophages, endothelial cells, etc.) reproducing inflammatory conditions by stimulation with cytokines or lipopolysaccharide (LPS) have previously been used to test the activity of various types of food extracts, semi-purified fractions or single purified bioactive molecules [57,58,59]. However, the gastro-intestinal mucosa is the principal tissue that interacts with the food matrix and intestinal bioavailability and metabolism represent important determinants for the activity of food bioactives [60,61,62]. The human intestinal Caco-2 cell line has extensively been employed over the last 20 years as a reliable in vitro tool for predicting intestinal absorption and metabolism of nutrients and drugs. Differentiated Caco-2 cells have also been used to assess the bioactivity of apple peel and cranberry phenolic fractions to counteract oxidative (200 μM Fe-ascorbate for 6 h) [32] or inflammatory stress (200 μM LPS for 6 h) [33], respectively. Similarly, the ability of *Artemisia annua* tea infusions [63] and of single purified phenolic compounds [64] to modulate the response to a cocktail of pro-inflammatory substances was tested on differentiated Caco-2 cells. We previously described a new Caco-2 cell inflammation model, based on the observation that depletion of intracellular zinc, caused by application of the zinc chelator TPEN, affects the response to the inflammatory citokine TNFα and shifts intestinal cell fate from survival to death [25]. Interestingly, clinical observations indicate that zinc supplements ameliorate Crohn’s disease symptoms and decrease intestinal permeability in experimental colitis, suggesting that the TPEN/TNFα Caco-2 cell inflammation model [25] reproduces mechanisms occurring in vivo, and may thus have physiological relevance. We therefore took advantage of this experimental model to compare the bioactivity of basal and enriched broccoli sprout juices. Pre-incubation with both juices ameliorated the loss of epithelial cells’ integrity induced by TPEN/TNFα, indicating that pre-incubation with the juice increased the cellular capacity to respond to an inflammatory stress. In addition, we report that juices produced from elicited sprouts were more effective in providing cell protection. Interestingly, broccoli sprout juices, prepared under the same controlled conditions used in this study, were also shown to be protective in a cellular model of Alzheimer’s disease and in Spontaneously Hypertensive Stroke Prone rats [65,66].

In the field of food science and technology, multivariate statistical analysis has been applied to study the quality, authenticity and geographical origin of different food samples [67], and has recently proved useful for the analysis of fruit juices [68]. By using PLSR, we developed a model to correlate cell protection (expressed as maintenance of high TEER) and bioactive content in our experimental system. This model successfully discriminated juice samples obtained from broccoli sprouts grown in different conditions (BJ vs. EJ) and highlighted reproducibility of independent juices prepared from sprouts grown under the same conditions. In addition, the model identified important phenolic compounds, including quercetin-3-Glc, cryptochlorogenic, neochlorogenic and cinnamic acid and several anthocyanins that significantly correlated to the cell protection effect. Interestingly, anthocyanins have been associated with anti-inflammatory effects both in vivo and in vitro, due to the coordinated induction of the expression of enzymes involved in both cellular antioxidant defenses and attenuation of the inflammatory response [6,69,70]. Moreover, quercetin glycosides and cinnamic acids have also been described to possess anti-inflammatory, and antioxidant properties in different experimental models [6,58,71]. SFN, frequently invoked to be the principal bioactive compound in broccoli [16,17], was present at similar levels in EJ and BJ and did not appear to correlate with the protective efficacy of the juices in our inflamed Caco-2 model. However, a contribution of SFN in this cell system cannot be excluded as it has been shown that it can act synergistically with quercetin on gene regulation [72], and that the combination of these two substances is more effective than either compound alone, in different cell lines [73,74,75].

## 4. Materials and Methods

### 4.1. Broccoli Sprouts’ Growth and Juice Preparation

Broccoli seeds (*Brassica oleracea* L. var. *botrytis* subvar*. cymosa*) were purchased from SUBA & UNICO (Longiano, FC, Italy). Seeds were surface sterilized by soaking for 15 min in 2% sodium hypochlorite under shaking, then drained and rinsed 10 times with distilled water. After soaking in distilled water for 16–18 h at 21 °C, seeds were rinsed in distilled water and transferred in the germination cylinder of Vitaseed sprouter (SUBA & UNICO) filled with distilled water. Sprouts were grown at 21 °C and 70% humidity in a plant growth chamber (Weiss Gallenkamp, Loughborough, United Kingdom) equipped with PHILIPS Master TL-D 36W/840 cool-white fluorescent tubes providing a photosynthetic photon flux density of 110 mmol m^−2^ s^−1^. Sprouts were grown in the dark for 5 days (basal condition). For the enriching condition, sprouts were grown under a long day (16 h light/8 h dark cycle) light regime and, after 3 days, water was replaced by 176 mM sucrose and growth was continued for two more days. The 5-day-old sprouts were rapidly but gently collected from the germination cylinder, weighted and immediately squeezed with a mechanical press (Angel 8500S, Living Juice srl, Lecco, LC, Italy). The juice was collected in ice-cold tubes, cleared by centrifugation (30 min, 3300× *g*, 4 °C) and aliquots were immediately frozen in liquid nitrogen and stored at −80 °C until further analysis. Three batches of each type of juice obtained from three independent sprout growths (biological replicates) were used for all subsequent analysis.

### 4.2. Chemicals

Solvents used for extraction and the high-performance liquid chromatography (HPLC)-grade methanol were of high purity (Carlo Erba, Milano, Italy). HPLC-grade water (18 mΩ) was prepared using a Millipore (Bedford, MA, USA) Milli-Q purification system. Folin and Ciocalteau’s phenol reagent, gallic acid, (+) catechin, aluminum chloride, sodium nitrite, HPLC-grade acetonitrile and formic acid were from Sigma-Aldrich (St. Louis, MO, USA).

### 4.3. High-Resolution Untargeted Analysis

Untargeted metabolomic analysis was performed according to the method of Rochfort et al. with minor modification [76]. The analysis was performed using a Dionex Ultimate 3000 (Thermo Scientific, Waltham, MA, USA) chromatographic system coupled with LTQ Orbitrap XL (Thermo Scientific). Opportunely diluted samples were injected into a Synergi Fusion 2.0 × 100 mm, 2.5 μm column (Phenomenex, Torrance, CA, USA) protected by Security Guard ULTRA UHPLC C18, 2.1 mm precolumn, at flow rate of 0.4 mL/min. Mobile phase A was water containing 0.1% formic acid, while mobile phase B was acetonitrile containing 0.1% formic acid (phase B). Elution gradient was: 95% A the first minute, 55:45 (A:B) in 12 min, from 55:45 (A:B) to 20:80 (A:B) in 2 min. The column was kept at 30 °C. Mass spectra were registered in positive and negative ion mode using resolving power for MS scan 30,000. Capillary temperature of electrospray was 320 °C, sheath gas flow 35 and auxiliary gas flow 5. Source voltage was 3.5 kV and 5.0 kV for negative and positive mode, respectively. Data were processed using Sieve 2.0 (Thermo Scientific) software. Framing was set to 10 ppm mass window ranging from 50 to 700 Da. Time width of the frames was 1 min ranging from 0 to 20 min. In order to optimize processing time and computer processing power, the maximum of frames was set to 5000.

### 4.4. Total Polyphenol, Flavonoid, and Anthocyanin Content

The total polyphenol content was determined by the Folin-Ciocalteu method using gallic acid for calibration curve and absorbance values of samples were converted to gallic acid equivalents (GAE) [77].

The total flavonoid content was determined by using a colorimetric method described previously [78]. (+)Catechin was used as reference compound for calibration curve and absorbance values of samples were converted to catechin equivalents.

The total anthocyanin content was determined by a spectrophotometric method according to Rapisarda et al. [79]. An aliquot of juice (0.1 mL) was diluted to 1.4 mL using a 80/20 (*v*/*v*) mixture of 95% ethanol and 37% HCl. Absorbance of resulting solution was measured at 420, 530, and 620 nm against a blank. Concentration of anthocyanins was calculated by the equation C (mg/L) = Net Abs_530_/slope × DF where Net Abs_530_ is given by Abs_530_ − (Abs_420_ + Abs_620_)/2, slope is the angular coefficient of a calibration line of Net Abs_530_ of standard solutions of cyanidin-3-glucoside in 80/20 solvent mixture, and DF is the dilution factor.

### 4.5. Phenolics Profiling

Samples of broccoli sprout juices were thawed on ice and diluted with methanol (1:1). Rosmarinic acid (RA) and malvidin 3-glucoside (Mal 3-Glu) were used as internal standards, and final concentrations were 2 mg/L and 10 mg/L, respectively. Samples were sonicated for 10 min, centrifuged for 10 min at 10,000 RPM, filtered over 0.22 mm Polyvinylidene Fluoride (PVDF) filters and injected into chromatographic system.

In order to quantify different phenolic compounds, a target metabolomic method was used using an Acquity UPLC connected to a Xevo TQMS (Waters, Milford, MA, USA) [80]. Reversed phase separation was performed with 100 mm × 2.1 mm, 1.8 μm column (Acquity HSS T3, Waters), protected with an Acquity UPLC HSS T3 1.8 mm precolumn (Waters). Mobile phases were composed of 0.1% formic acid (FA) in water (phase A) and 0.1% of FA in Acetonitrile (phase B). Flow was set at 0.4 mL/min. Linear gradient started from 5% B to 20% B in 3 min, followed by isocratic step at 20% B for 1.3 min and two additional steps, from 20% to 45% B in 4.7 min and from 45% to 100% B in 2 min. Mass spectrometry detection of phenols was performed with electrospray ionization (ESI) in positive and negative modes as described by Vrhovsek et al. [80].

For the detection of anthocyanins, we followed the method of Arapitsas et al. with minor modification [81]. The analysis was performed using an Acquity UPLC connected to a Xevo TQMS, equipped with an Acquity UPLC BEH C18 1.7 μm, 2.1 mm× 150 mm column (Waters), and an Acquity UPLC BEH C18 1.7 mm procolumn (Waters). Mobile phases were composed of 5% FA in water (A) and 5% FA in methanol (B). Identification of individual anthocyanins was based on MRM transitions and retention times as previously reported [51,81]. Results were expressed as cyanidin 3,5-diglucoside equivalents. 

Processing of raw data sets was performed with the help of Mass Lynx Target Lynx Application Manager (Waters).

### 4.6. Glucosinolate, Sulforaphane and Sulforaphane Nitrile Determination

GLSs were determined in broccoli sprout juices by a HPLC MS/MS method according to Maldini et al. [27]. Fourteen GLSs were analyzed, namely gluconapin, progoitrin, sinigrin, gluconapoleiferin, glucoraphanin, glucoiberin, glucoerucin glucocheirolin, glucoiberverin, glucoalysin, 4-methoxyglucobrassicin, neoglucobrassicin, 4-hydroxyglucobrassicin, glucobrassicin. Sulforaphane (SFN) determination was performed using an HPLC system (Perkin-Elmer, Waltham, MA, USA) interfaced with an Applied Biosystems (Foster City, CA, USA) API3200 Q-Trap spectrometer. Quantitative on-line HPLC-ESI-MS/MS analyses were performed using mass spectrometer in negative (for GLSs) and in positive (for SFN and SFN-nitrile) Multiple Reaction Monitoring (MRM) mode. The API 3200 ES source was tuned by infusing a standard solution of SFN (1 μg/mL in methanol 50%) into the source at a flow rate of 10 μL/min. The optimized parameters were: declustering potential 45 eV, entrance potential 5 eV, collision energy 18 eV; fragmentation reactions selected for SFN and SFN-nitrile were 178→14 (CE = 18; CXP = 4; CEP = 14) and 146→7 (CE = 25; CXP = 4; CEP = 13), respectively. The source temperature was held at 400 °C and the voltage applied was 5500. The dwell time was 120 ms.

Juice samples were opportunely diluted in H_2_O with 0.1% formic acid, filtered, injected (10 μL) into a Luna C18 column (Phenomenex, Torrance, CA, USA) (150 × 2.1 mm i.d., 5 µm) and eluted at flow rate of 0.3 mL/min. Mobile phase A was H_2_O containing 0.1% formic acid while mobile phase B was acetonitrile containing 0.1% formic acid. Elution gradient was: 100% A, 20:80 (A:B) in 20 min, from 20:80 (A:B) to 0:100 (A:B) in 1 min. The column was kept at 25 °C, using a Peltier Column Oven Series 200 (Perkin Elmer). Data acquisition and processing were performed using Analyst software 1.5.1. Both SFN and SFN-nitrile concentration was calculated over an external standard curve of SFN.

### 4.7. Cell Culture

The Caco-2 cell line, obtained from INSERM (Paris, France), was routinely sub-cultured at 50% density [31], and was maintained at 37 °C in a 90% air–10% CO_2_ atmosphere in Dulbecco Minimum Essential Medium (DMEM) containing 25 mM glucose, 3.7 g/L NaHCO3, 4 mM l-glutamine, 1% nonessential amino acids, 100 U/L penicillin, 100 μg/L streptomycin (complete medium), supplemented with 10% heat-inactivated fetal bovine serum (FBS Hyclone Laboratories, Logan, UT, USA). All reagents were from Sigma-Aldrich (Milan, Italy).

For differentiation, cells were seeded on polycarbonate filters, 12 mm diameter, 0.4 μm pore diameter (Transwell, Corning Inc. Lowell, MA, USA) at a density of 3.5 × 10^5^ cells/cm^2^ in complete medium supplemented with 10% FBS in both AP and BL compartments for two days to allow the formation of a confluent cell monolayer. From day 3 after seeding, cells were transferred to complete medium in both compartments, supplemented with 10% FBS only in the BL compartment and allowed to differentiate for 21 days with regular medium changes three times a week [30].

### 4.8. Measure of Monolayer Integrity

To determine the effects of the treatments on the permeability of intestinal tight junctions and the integrity of the cell monolayer in Caco-2 cells, Trans-Epithelial Electrical Resistance (TEER) was measured at 37 °C using the voltmeter apparatus Millicell (Millipore , Merck Group, Darmstadt, Germany) provided with Ag/AgCl electrodes, as previously described [82]. TEER was expressed as Ω·cm^2^ = (Ω cells − Ω filter) · A, where Ω cells is the monolayer resistance, Ω filter is the resistance of the filter by itself and A is the filter area (cm^2^).

### 4.9. Experimental Intestinal Cell Model

Prior to each experiment, differentiated Caco-2 cells were pre-incubated for 14 h in DMEM without addition of FBS (experimental medium), with or without 250 μL/mL sprout juice in the apical (AP) compartment. To achieve marginal zinc depletion, cells were incubated in experimental medium containing 20 μM *N*,*N*,*N*′,*N*′-tetrakis (2-pyridylmethyl) ethylene-diamine (TPEN) (Sigma-Aldrich Co., Milan, Italy) for 2 h. Following TPEN removal, they were exposed to fresh medium containing 2 ng/mL TNFα (Sigma-Aldrich Co., Milan, Italy), as previously described [25].

### 4.10. Statistical Analysis

All the analyses were performed using three juices for each condition, obtained from independent sprout growths (biological replicates). All analytical measurements and cell culture experiments were performed in triplicate and statistical analyses were performed using Microsoft Office Excel 2011 upgraded with XLSTAT (ver. 4 March 2014). Data were expressed as mean ± SD and analyzed by one-way ANOVA followed by Fischer *post hoc* test.

For multivariate data analysis, Partial Least Square Regression (PLSR) was performed with Unscrambler v 10.2 (CAMO Software AS, Oslo, Norway) using the Non-Linear Iterative Partial Least Squares (NIPALS) algorithm. Targeted chemical data constituted the independent X-block of variables, while the cell protection attributes represented the dependent Y-variable. Data were normalized by mean centering using the 1/(standard deviation) transformation to ensure that all variables had equal potential influence. The calibration model was validated by full cross-validation. Weighted regression coefficients (BW) for the relationships between chemical and biological variables were determined by applying Martens’ uncertainty test option. Throughout all data analysis, effects were considered to be significant at a level of *p* < 0.05.

## 5. Conclusions

*Brassica oleracea* sprout juices were shown to confer protection to an in vitro model of inflamed human intestinal epithelium based on Caco-2 cells treated with TNF-α under marginal zinc deprivation. In addition, nutraceutical improvement of the juice obtained through elicitation of the sprouts increased the protective potential of the juice. By combining controlled growth and metabolic profiling of the sprouts with multivariate analysis, we demonstrate that the protective effect is reproducible and is correlated to a number of bioactive molecules whose levels are indicative of a functional enrichment of the food matrix. While the phytochemicals that significantly correlated with the protective effect can be used as reliable descriptors of the biological potential of BJ and EJ juices, further investigation is needed to demonstrate that these molecules are causally related to the bioactivity observed in Caco-2 cells.

## Figures and Tables

**Table 1:** Targeted analysis of broccoli sprouts’ juice content.

		Basal Juice			Enriched Juice			Fold	
		(BJ)			(EJ)			(EJ/BJ)	
TP	Total Phenols (mg GAE/mL)	1.86	±	0.24	3.01	±	0.34	1.6	**
TF	Total Flavonoids (mg CE/mL)	0.50	±	0.08	1.29	±	0.30	2.6	*
TA	Total Anthocyanins (μg Cy_3_GlcE/mL)	4.29	±	0.48	40.15	±	15.28	9.4	*
SFN	Sulforaphane (μg/mL)	19.37	±	2.65	21.90	±	1.57	1.1	
SFNnit	Sulforaphane nitrile (μg/mL)	3.47	±	0.31	4.50	±	0.44	1.3	*
**Polyphenols** (μg/mL)									
4-ABA	4-Aminobenzoic acid	0.08	±	0.02	0.04	±	0.01	0.6	
CA	Caffeic acid	0.01	±	0.01	0.19	±	0.06	13.5	*
Cat	Catechin	2.37	±	0.24	3.06	±	0.20	1.3	*
ChlA	Chlorogenic acid	0.08	±	0.02	1.11	±	0.37	13.5	*
CinA	Cinnamic acid	0.01	±	0.00	0.04	±	0.01	3.5	**
ConAl	Coniferyl alcohol	0.17	±	0.01	0.39	±	0.13	2.4	*
pCouA	p-Coumaric acid	0.29	±	0.06	1.54	±	0.47	5.2	*
CryChlA	Cryptochlorogenic acid	0.02	±	0.01	0.15	±	0.03	7.1	**
FA	Ferulic acid	0.38	±	0.03	0.64	±	0.17	1.7	
IsoR3Glc	Isorhamnetin-3-Glc	0.05	±	0.03	0.03	±	0.01	0.7	
NChlA	Neochlorogenic acid	0.24	±	0.02	12.03	±	1.96	49.5	***
ProCyB2	Procyanidin B2	0.02	±	0.01	0.04	±	0.01	1.7	
Q3Glc	Quercetin-3-Glc	n.d.			0.21	±	0.01		***
Q34diGlc	Quercetin-3.4-diGlc	0.15	±	0.05	0.88	±	0.34	5.9	*
SinA	Sinapic acid	38.01	±	12.02	25.59	±	7.12	0.7	
SinAl	Sinapyl alcohol	0.15	±	0.07	0.33	±	0.04	2.2	*
SyrAld	Syringaldehyde	0.05	±	0.01	0.02	±	0.01	0.4	*
SyrA	Syringic acid	0.41	±	0.02	0.28	±	0.06	0.7	*
**Anthocyanins** (μg/mL) ^1^									
Cy3_a	Cy_3_sinapoyl_sinapoyl_diGlc_5_malonyl_Glc	n.d.			0.46	±	0.11		**
Cy3_b	Cy_3_sinapoyl_feruloyl_diGlc_5_malonyl_Glc	n.d.			0.76	±	0.27		*
Cy3_c	Cy_3_coumaryl_synapoyl_diGlc_5_malonyl_Glc	n.d.			1.66	±	0.27		***
Cy3_d	Cy_3_sinapoyl_synapoyl_diGlc_5_Glc	n.d.			1.08	±	0.34		**
Cy3_e	Cy_3_coumaryl_feruoyl_diGlc_5_malonyl_Glc	n.d.			1.11	±	0.38		*
Cy3_f	Cy_3_sinapoyl_feruloyl_diGlc_5_Glc	n.d.			1.68	±	0.30		**
Cy3_g	Cy_3_sinapoyl_diGlc_5_malonyl_Glc	n.d.			0.37	±	0.08		**
Cy3_h	Cy_3_feruloyl_diGlc_5_malonyl_Glc	n.d.			0.29	±	0.01		***
Cy3_i	Cy_3_caffeyl_diGlc_5_malonyl_Glc	n.d.			0.07	±	0.06		
Cy3_l	Cy_3_coumaryl_diGlc_5_malonyl_Glc	n.d.			0.55	±	0.07		***
Cy3_m	Cy_3_sinapoyl_diGlc_5_Glc	n.d.			0.94	±	0.11		***
Cy3_n	Cy_3_feruloyl_diGlc_5_Glc	n.d.			0.85	±	0.20		**
Cy3_o	Cy_3_coumaroyl_diGlc_5_Glc	n.d.			0.84	±	0.08		***
Cy3_p	Cy_3_diGlc_5_Glc	0.58	±	0.05	1.61	±	0.25	2.8	**

Data are mean ± SD (*n* = 3); n.d. = not detected or values below LOD; ^1^ expressed as cyanidin 3.5-diglucoside equivalents; * *p* < 0.05; ** *p* < 0.005; *** *p* < 0.0005.

**Table 2:** Partial Least Square (PLS) regression model to explore the relationship between phytochemical content and protective effect on Caco2 cells of broccoli sprout juice samples.

Attributes	% Explained Variance		No. of Factors	Correlation ^a^		Validation ^a^		BW ^b^	
	X	Y		R^2^	RMSE	R^2^	RMSE	Positive	Negative
TEER	92%	100%	2	0.99	3.59	0.96	25.31	Cinnamic acid; Cryptochlorogenic acid; Neochlorogenic acid; Procyanidin B2; Quercetin_3_Glc; Cy_3_diGlc_5_Glc; Cy_3_sinapoyl_feruloyl_diGlc_5_Glc; Cy_3_feruloyl_diGlc_5_malonyl_Glc; Cy_3_coumaroyl_diGlc_5_Glc; Cy_3_coumaryl_diGlc_5_malonyl_Glc; Cy_3_coumaryl_synapoyl_diGlc_5_ malonyl_Glc;	Synapic acid

^a^ R^2^ = square of the Pearson correlation value; RMSE = Root mean square error; ^b^ List of chemical compounds with significant positive and negative weighted regression coefficients (BW) with TEER.
